# Malaria infected red blood cells release small regulatory RNAs through extracellular vesicles

**DOI:** 10.1038/s41598-018-19149-9

**Published:** 2018-01-17

**Authors:** Kehinde Adebayo Babatunde, Smart Mbagwu, María Andrea Hernández-Castañeda, Swamy R. Adapa, Michael Walch, Luis Filgueira, Laurent Falquet, Rays H. Y. Jiang, Ionita Ghiran, Pierre-Yves Mantel

**Affiliations:** 1 Department of Medicine, University of Fribourg, 1700 Fribourg, Switzerland; 2 Department of Global Health (GH) & Center for Drug Discovery and Innovation (CDDI), College of Public Health, University of South Florida, Tampa, FL 33612 USA; 3 Division of Allergy and Infection, Beth Israel Deaconess Medical Center, Boston, MA 02115 USA

## Abstract

The parasite *Plasmodium falciparum* causes the most severe form of malaria. Cell communication between parasites is an important mechanism to control population density and differentiation. The infected red blood cells (iRBCs) release small extracellular vesicles (EVs) that transfer cargoes between cells. The EVs synchronize the differentiation of the asexual parasites into gametocytes to initiate the transmission to the mosquito. Beside their role in parasite communication, EVs regulate vascular function. So far, the exact cargoes responsible for cellular communication remain unknown. We isolated EVs from cultured iRBCs to determine their small RNA content. We identified several types of human and plasmodial regulatory RNAs. While the miRNAs and tRNA-derived fragments were the most abundant human RNAs, we also found Y-RNAs, vault RNAs, snoRNAs and piRNAs. Interestingly, we found about 120 plasmodial RNAs, including mRNAs coding for exported proteins and proteins involved in drug resistance, as well as non-coding RNAs, such as rRNAs, small nuclear (snRNAs) and tRNAs. These data show, that iRBC-EVs carry small regulatory RNAs. A role in cellular communication is possible since the RNAs were transferred to endothelial cells. Furthermore, the presence of *Plasmodium* RNAs, in EVs suggests that they may be used as biomarker to track and detect disease.

## Introduction

Malaria affects over 400 million individuals worldwide every year, killing about 0.5 million^1^. The complications of the disease arise during the blood stage, which is characterized by symptoms such as cerebral malaria, severe anemia, metabolic acidosis and respiratory distress^2^. During the blood stage, the parasites release toxic factors that contribute to inflammation. In addition, iRBCs secrete small vesicles that contain human and parasite-derived biomolecules^3^. The EVs derived from iRBCs are elevated in patients suffering from malaria and are particularly elevated in severe disease^4^. Interestingly, the severity of the disease significantly correlates with the level of EVs in the plasma^5^. Therefore, EVs might be used as biomarker to track the progress of the disease and treatment. EVs of a size between 100–300 nm have been characterized to contain both human and parasite proteins^3,6^.

Surprisingly, EVs induce the conversion of parasites into gametocytes^3^. Thus, EVs act like a master regulator that orchestrate and synchronize the conversion to gametocytes to optimize the transmission of the parasites to mosquito. Although the molecules responsible for the commitment have not yet been identified, EVs transfer nucleic acids from parasite to parasite as demonstrated by the transfer of a plasmid DNA encoding for a gene conferring resistance to a particular drug^6^. Only the parasites that have acquired the selection marker can grow under the drug selection pressure. While these experiments demonstrated the potential of EVs to carry and transfer functional nucleic acids between parasites, the authors characterized for the first time a mechanism of communication between parasites. Interestingly malaria EVs contain RNAs, in particular small RNAs^7^. However so far, little is known about the nature of these RNAs.

During malaria, host derived miRNAs constitute essential mediators of cellular communication. In fact, functional miR451a, a miRNA expressed in RBCs is transferred from iRBCs to endothelial cells via EVs. Remarkably, miR451a is bound to Argonaute-2 and forms a functional silencing complex. Once transferred, miR451a destabilizes specific mRNA to regulate endothelial cell barrier function^7^.

Besides miRNAs, many other small RNA species have regulatory functions. However so far most of the analysis of EV RNAs have focused on miRNAs. High-throughput RNA-Seq has allowed the identification of new small RNAs with regulatory properties. Such as tRNA-derived fragments, piwi-RNAs (piRNAs), Vault RNAs (vtRNAs), Y RNAs and snoRNAs^8,9^. Furthermore, *P. falciparum* might use EVs to shuttle its own plasmodial RNAs. Currently no study has directly addressed the RNA composition of EVs.

Here we collected EVs derived from *in vitro* cultures of *P. falciparum* and performed RNA-Seq to characterize for the first time the small RNA content of iRBCs derived EVs. We found several species of host as well as plasmodial RNAs. Remarkably, EVs transferred *Plasmodium* RNAs to endothelial cells. While our data highlight a rich repository for infection-related biomarkers, we also identify an entirely new strategy by which *Plasmodium* likely manipulates host defensive barriers. Further exploration of EV functions may identify novel strategies for malaria control.

## Results

### Characterization and properties of extracellular vesicles isolated by differential centrifugation

**Figure 1:**
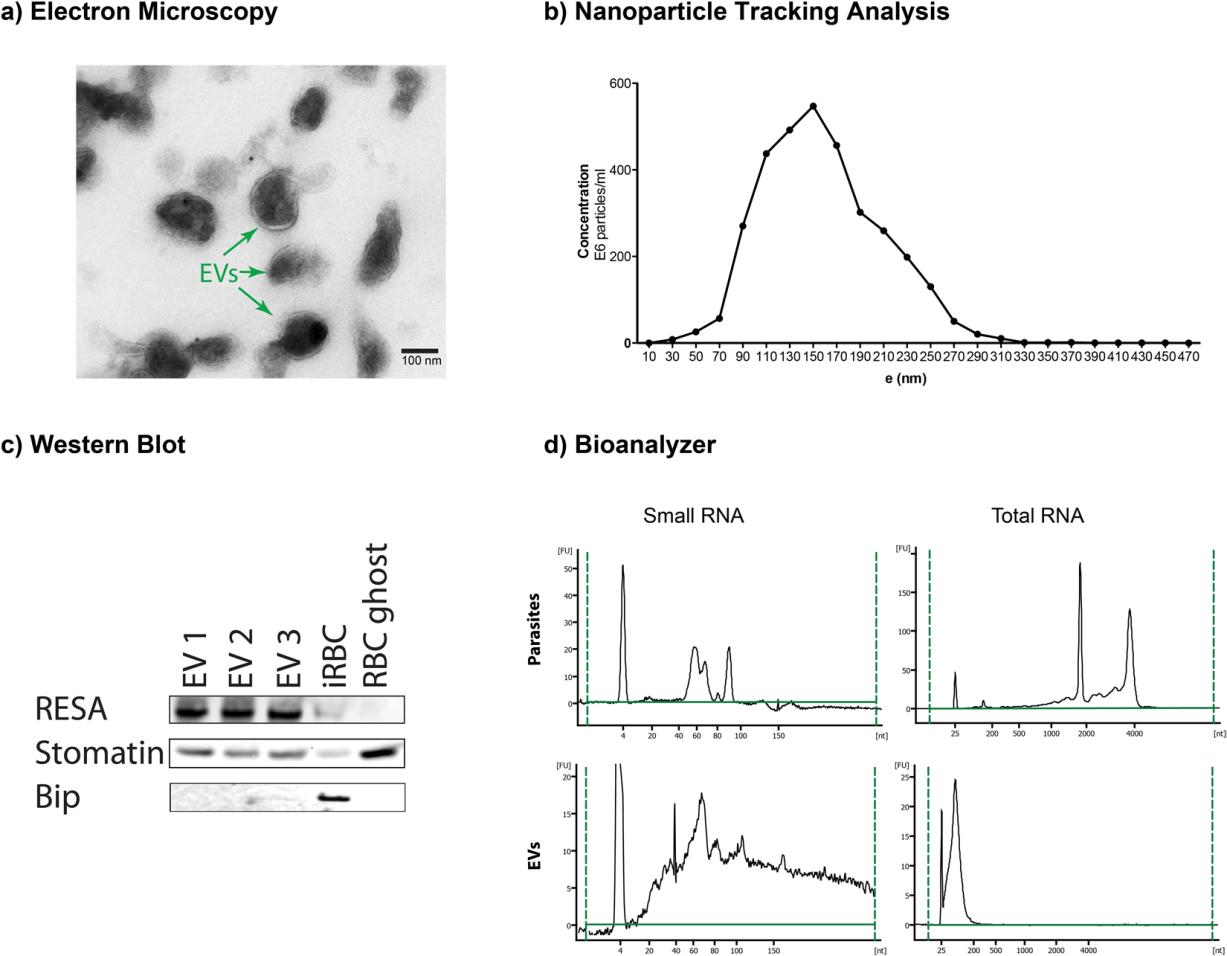
Extracellular vesicles isolated from *P. falciparum* carry RNA. (**a**) Transmission electron microscopy of EV preparation, purified EVs show individual EVs and a few clumps of varying sizes and intact lipid bilayers. The scale bar is 100 nm. (**b**) Representative particle size distribution from purified EVs. The concentration and size are determined by Nanoparticle Tracking Analysis. One representative experiment is shown. (**c**) Isolated EVs were confirmed by Western Blot for the enrichment of EV markers stomatin and RESA and negative for the endoplasmic reticulum Bip. (**d**) Total and Small bioanalyzer profiles of EV RNAs and total cell RNA show enrichment of small RNAs in EVs. One representative experiment is shown.

To further investigate the potential regulatory RNAs present in EVs, we collected EVs from *P. falciparum* iRBCs cultures and purified the vesicles by ultracentrifugation. To verify the vesicular structure of the isolated EVs, we used transmission electron microscopy (TEM). Analysis by TEM revealed that EVs have a size of 100–250 nm and demonstrated an intact lipid bilayer (Fig. 1a). The morphology is consistent with EVs as previously reported measuring about 150 nm in size as observed by TEM and the nanoparticle tracking system (Fig. 1b). Larger EVs observed by the nanoparticle tracking system might be the result of aggregate formation^3^. The purity of the EV preparations was determined by western blotting with a panel of antibodies. EVs were enriched in the lipid raft protein stomatin as well as the parasite protein RESA, while Bip a plasmodium protein specifically expressed in endoplasmic reticulum was absent (Fig. 1c).

To further define the EV cargoes, we isolated total RNA from EVs and intact iRBC cells. The quantity and quality of isolated RNA were determined using an Agilent Bioanalyzer. In contrast to intact cells, total RNA bioanalyzer profiles indicated that iRBC EVs contain minor amounts of 18 S and 28 S rRNA species. Furthermore, the RNA composition is different in EVs in comparison with intact cells, with enrichment in small RNAs below 300 nt in length in the vesicles (Fig. 1d).

### Extracellular vesicles derived from iRBCs contain small RNAs

Next to decipher the RNA content of EVs, we generated small RNA libraries of the samples for small RNA-Seq from three biological replicates of EVs derived from iRBCs. The same 3D7 parasite strain was cultured in RBCs of three different donors.

**Figure 2:**
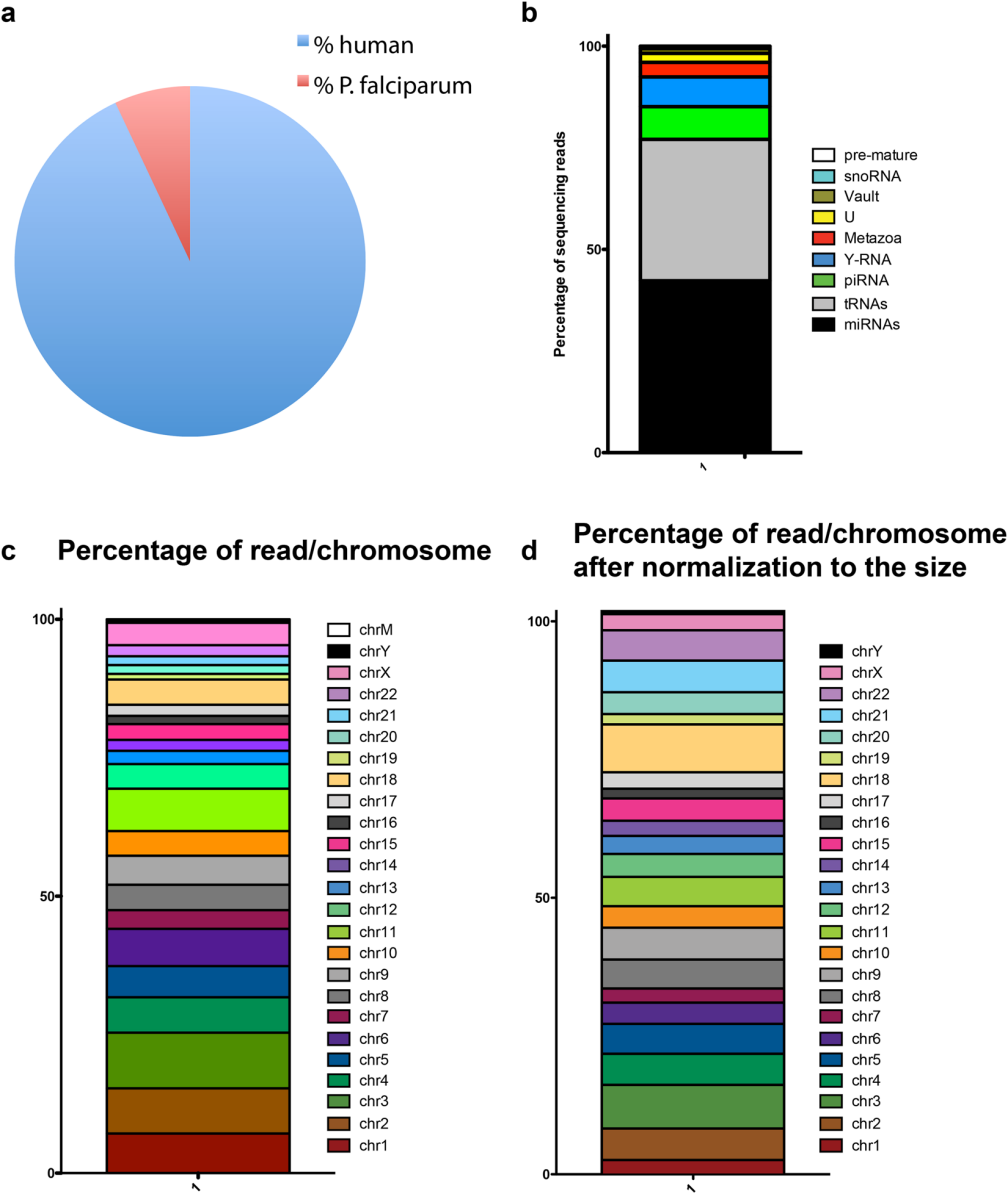
Characterization of small RNAs by NGS. (**a**) Composition of reads mapping to human and *P. falciparum* genomes. (**b**) Distribution of iRBC EV small RNAs. Segments of the bar indicate the percent of reads attributed to each RNA biotype among all RNA reads that mapped perfectly to known sequences, averaged across 3 biological replicates is shown. (**c**) Reads aligning to coding genes were mapped back to the human chromosomes and represented as the % reads aligning to each chromosome. (**d**) Reads aligning to coding genes were mapped back to the human chromosomes and after normalization to the nucleotide size of the chromosome.

Small RNA sequencing of the EV libraries yielded a total of 83’319’134 raw reads that were pre-processed and trimmed to remove adapter sequences using sRNAbench to identify high quality reads that were considered for further analysis. After processing for adapter and unmapped sequences, the number of reads was reduced to 26’647’563, 8’126’655 and 11’925’918 respectively. The total number of reads mapping to the human genome was 43’689’670, whereas the total number of reads mapping to the *P. falciparum* genome was 3’010’466. Therefore, the proportion of *P. falciparum* reads was 6.4% versus 93.6% of human (Fig. 2a and Table 1).

**Table 1.** Sequencing statistics.

	Sample 1	Sample 2	Sample 3	Average
Sequencing reads	68003555	33846106	35423492	45757717
QC passed reads	51798945	14610329	16909860	27773044
Mapped to human genome	24968040	7274347	11447283	14563223
Mapped to plasmodium falciparum genome	1679523	852308	478635	1003488
Identified miRNAs	219	87	276	

Since small RNAs have regulatory functions, we focused our analysis on the identification of small RNAs. As expected miRNAs and tRNAs were widely and abundantly expressed. Then we performed a detailed quantification of annotated RNAs to functionally categorize the human small RNAs. The average across all three samples was calculated to obtain a representative profile of biological diversity between donors. Of total reads, the miRNAs were the most abundant small RNA species with 42.3%, followed by 34.79% of tRNAs, 8% piRNA, 7% Y RNA, 3.60% Metazoa Signal Recognition Particle (SRP) RNA, 2.17% U-RNA, 1.17% Vault RNA, 0.48% snoRNA and 0.14% pre-mature RNA (Fig. 2b). The reads map to all the human chromosomes in particular chromosome 1 and 3 (Fig. 2c). Since the chromosomes do not have the same size, we then normalized by size to look for specific enrichment. Since there are several copies of mitochondrial DNAs per cell, we excluded them from the analysis. After normalization, there was an enrichment in chromosome 18 and 22 (Fig. 2d).

### EVs contain a subset of human regulatory miRNAs

**Figure 3:**
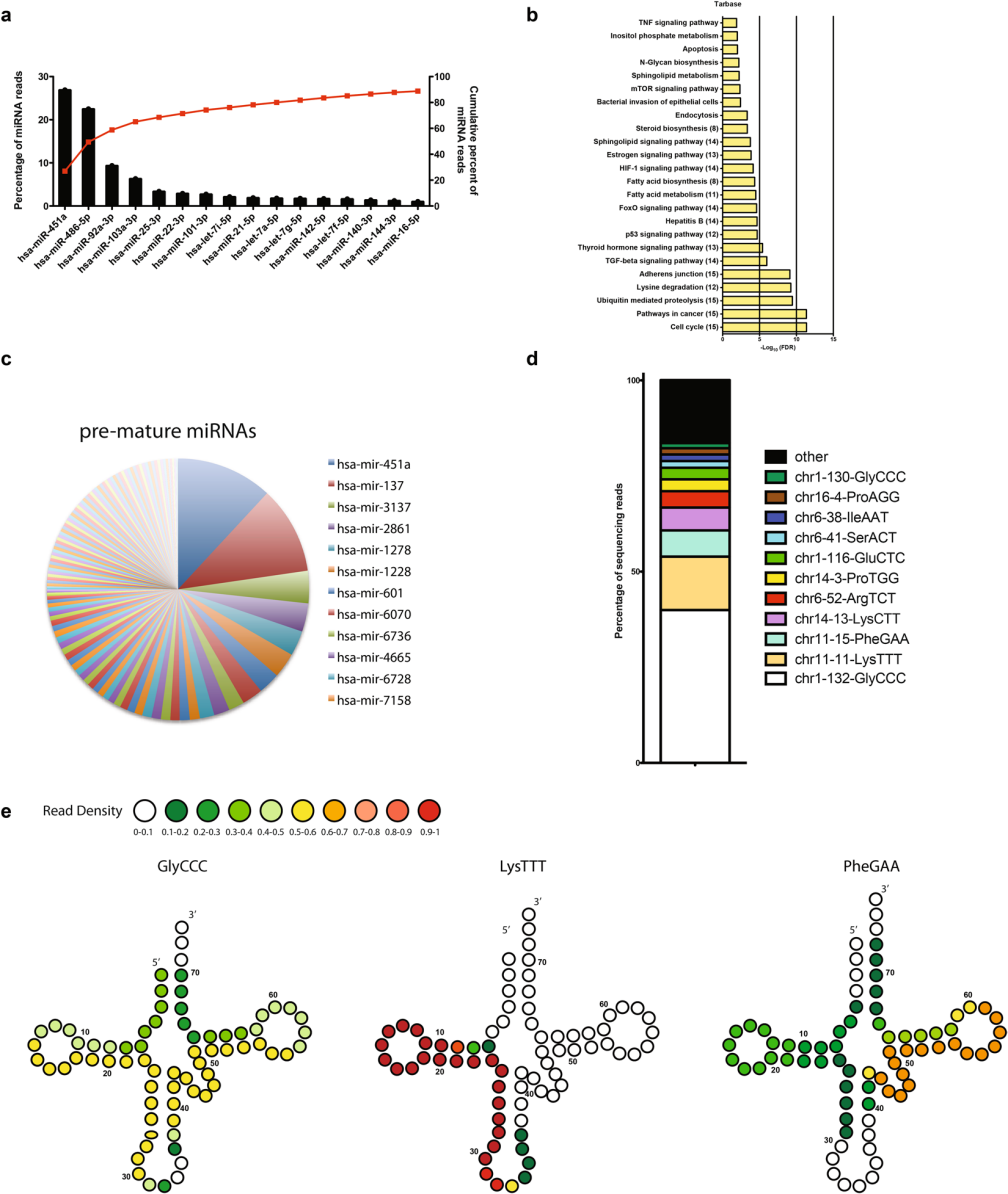
Profiling of human miRNAs and tRNAs isolated from *P. falciparum* infected EVs. (**a**) Top 16 most highly expressed unique miRNAs in EVs. Left axis and bars: percent of total miRNA reads that mapped to each of the top 16 most abundant miRNAs. Error bars are standard deviation (n = 3). Right axis and line: cumulative percent of total miRNA reads. (**b**) Significantly (Benjamini Hochberg corrected P value < 0.05) enriched KEGG pathways for target genes. The number of miRNA involved in targeting at least one gene in a given pathway are shown in parentheses. (**c**) Distribution of human pre-mature miRNAs (n = 3). (**d**) Distribution of known tRNA sequences based on the total read counts. tRNA sequences are categorized by number of read counts corresponding to each individual tRNA (n = 3). (**e**) Based on the small RNA-seq results, only tRNA fragments were identified in EVs. Shown are examples of tRNA structures for each species depicting the boundaries of the identified tRNA fragment. The read density at each position is shown as a heatmap.

A total of 305 human miRNAs were identified in at least one sample. While we identified many miRNAs, only a few sets were highly expressed^7^ (Fig. 3a). A total of 61 miRNAs were present in all the 3 samples and among them, miR451a was the most abundant (515 RPM) accounting for 26.9% of the total miRNA reads, followed by miR486-5p (22.51%), miR92a-3p (9.37%) and miR103a-3p (6.34%). The most abundant miRNAs represent over 65% of the total reads (Fig. 3a). Because only a few miRNA species accounted for the majority of miRNA sequencing reads, we focused on the top 16 miRNAs for our analysis of potential regulatory targets. We performed Kyoto Encyclopedia of Genes and Genomes (KEGG)^10^ pathway enrichment analysis of genes targeted by miRNAs relative to all target genes in miRDB (http://www.genome.jp/kegg). We found that pathways involved in cell cycle, cell adhesion and associated to cancer were significantly enriched (Fig. 3b). In addition to mature RNAs, we found pre-mature miRNAs. However, the RPM for the pre-mature miRNAs were low and only 3 had an RPM above 1. In fact, pre-mature miR451a and miR137 were the highest expressed with 3.4 and 1.5 RPM respectively. Although the abundance was low, they were expressed in all three preparations (Fig. 3c).

The human tRNAs represented an abundant small RNA category in our samples. We found that 60 tRNAs were expressed out of which 42 tRNAs were expressed in all the three biological replicates. However over 75% of the reads were derived from tRNA-GlyCCC, tRNA-LysTTT, tRNA-PheGAA and tRNA-LysCTT. With an average of 615 RPM, tRNA-GlyCCC represents about 44% and tRNA-LysTTT 16% of the total tRNA reads (Fig. 3d). All the tRNAs identified were tRNA fragments, we did not discover full length tRNAs. We found the 3′ and 5′ halves of tRNA –GlyCCC, with each half consisting of about 32 nt. The 5′half was slightly more abundant than the 3′. In addition, we found 5′tRNA-LysTTT of about 24 nt. Finally, tRNA-PheGAA, was composed of a 5′tRNA fragment and a 3′ fragment of about 24 nt each (Fig. 3e and Supplementary Figure 1).

### Extracellular vesicles contain a subset of Y-RNA, Vault RNA, SRP-RNA, U RNA, piRNA and snoRNAs

Although miRNAs and tRNAs have essential biological functions, other small non-coding RNAs have in general not been characterized in EVs. Our sequencing strategy favors the identification and quantification of additional RNAs. Apart from the miRNAs and tRNA fragments, other RNA biotypes were abundantly present in iRBC-EVs, including Y-RNA, vault-RNA, SRP-RNA (7SL-RNA), U-RNA, Piwi-RNA and sno-RNA.

**Figure 4:**
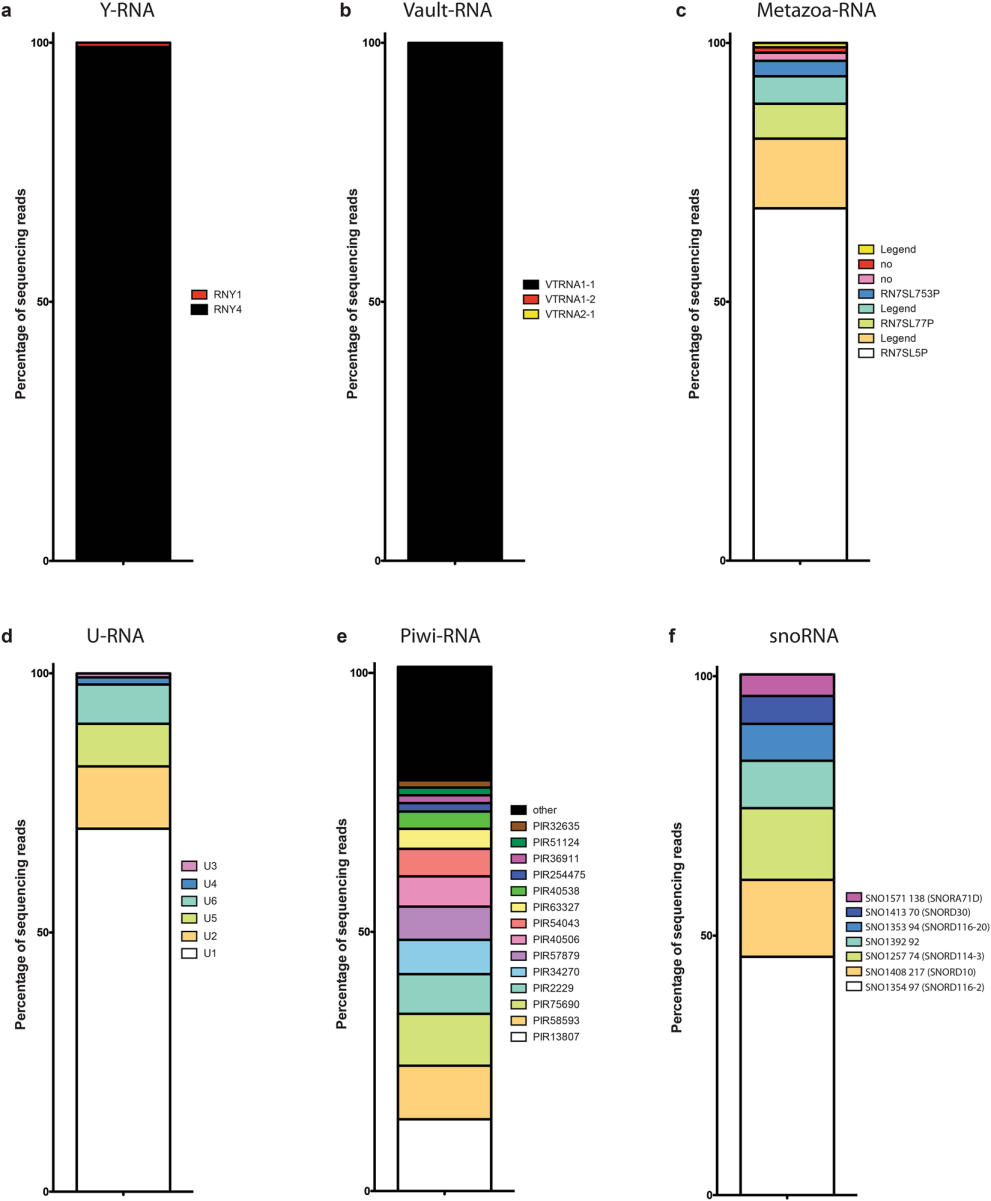
Functional characterization of small RNAs. (**a**) Distribution of Y-RNA (**b**) Vault-RNA. (**c**) Distribution of Metazoa-RNA. (**d**) Distribution of U-RNA. (**e**) Distribution of PIWI-RNAs. (**f**) Distribution of snoRNAs.

Y-RNAs, which range in size from 83 to 112 nt, are cytoplasmic non-coding RNAs that are conserved in the animal kingdom^11–13^. Most Y-RNAs are bound to the proteins Ro60 and La to form Ro ribonucleoproteins (RNPs), which might be targeted by the immune system in autoimmune disease^14^. There are four non-coding Y-RNAs in humans (hY1, hY3, hY4 and hY5 RNAs). We identified 2 Y-RNAs in our samples, RNY4 (319.37 RPM) and RNY1 (3.2 RPM), (Fig. 4a). Therefore, RNY4, which was identified in all 3 replicates accounted for 99% of the RPM for the total Y-RNAs^15^.

Vault RNAs are the RNA components of Vault ribonucleoprotein particles, which are located in the cytoplasm. The three human vRNA genes contain 88 (vRNA2 and vRNA3) or 98 nucleotides (vRNA1) single-exon polymerase III transcripts^16^. A fourth vRNA-related sequence, vRNA4, presumed to be silent pseudogene is located on chromosome X^16^. Only 1 Vault RNA was identified, VTRNA1-1 (51.31 RPM) having the highest level of expression and representing 99.5% of the vRNA reads (Fig. 4b).

Next, we identified 8 different SRP-RNAs, with 4 of them expressed in all three samples. RN7SL5P was the most abundant with an average RPM count of 107, which represents 68% (Fig. 4c) of all the SRP-RNAs. SnRNAs, also commonly referred to as U-RNA are highly conserved non-coding RNAs located in the nucleus and associated with Sm ribonucleoproteins as well as other specific proteins, to form small nuclear ribonucleoproteins. They participate in the splicing of precursors mRNAs. SnRNAs have an average size of 150 nt. We identified a total of 6 different U RNA (U1, U2, U3, U4, U5 and U6) with U1 being the most abundant, representing 70% of the total counts (Fig. 4d).

Piwi-associated RNAs, as compared with miRNAs, are a distinct class of 24–31 nucleotide-long RNAs produced by a Dicer-independent mechanism from single-stranded precursor transcripts expressed from intergenic regions termed piRNA clusters^17^. In total, we detected 44 piRNAs of which 21 were expressed in all three samples. The three most abundant piRNAs (PIR13807, PIR58593 and PIR57690) together represent approximately 33% of the total piRNA reads (Fig. 4e).

Small nucleolar RNAs (snoRNAs) are a class of small RNA molecules that primarily guide chemical modifications of other RNAs, mainly ribosomal RNAs and small nuclear RNAs^18^. We identified 7 snoRNAs with a total of 35 RPM (Fig. 4f). SNORD116-2 was the most abundant with 16 RPM and 46% of the total count.

### *P. falciparum* iRBCs release plasmodial RNAs

**Figure 5:**
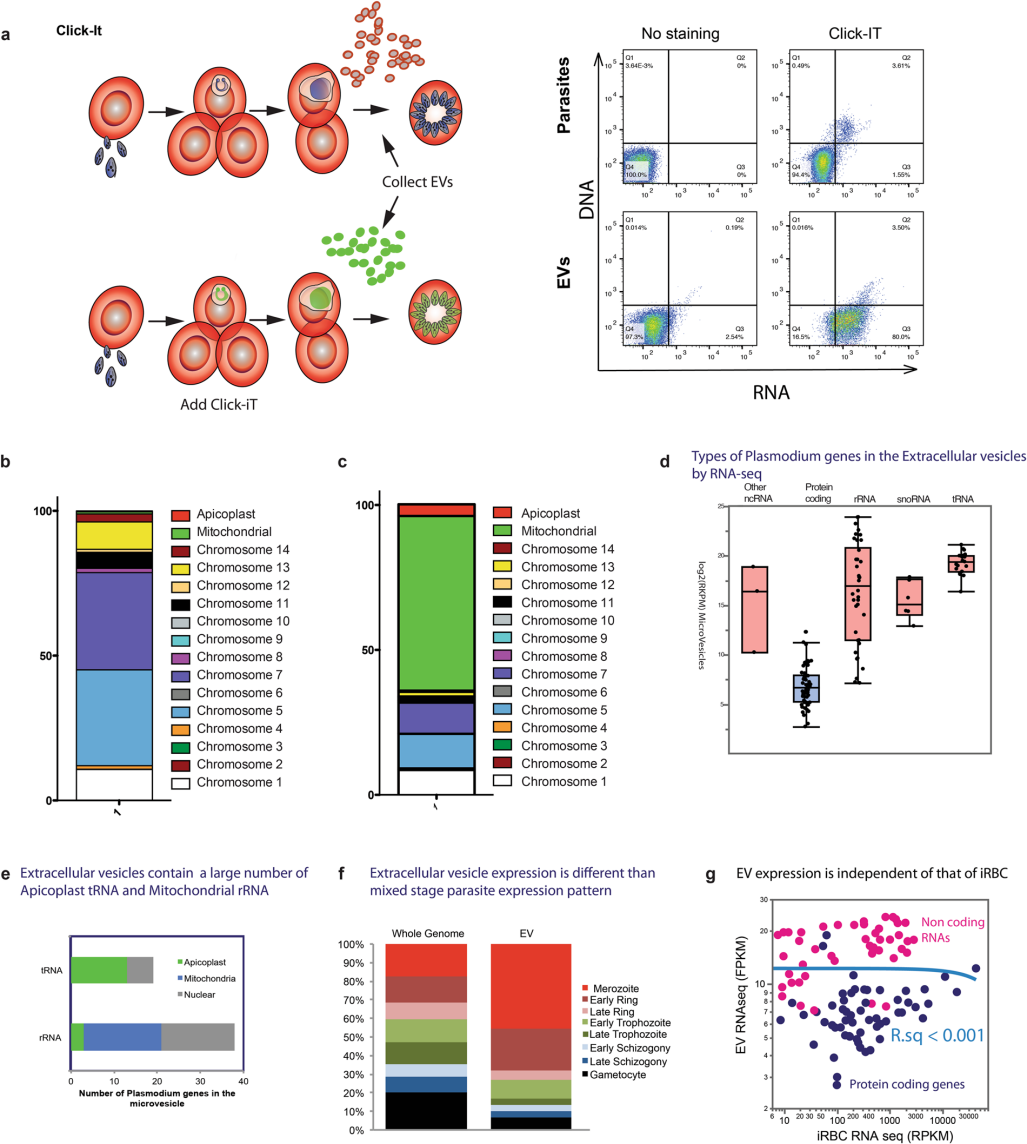
EVs contain plasmodial RNAs. (**a**) Quantification of *P. falciparum* RNAs transferred to EVs. Infected RBCs were labeled with Click-It RNA Alexa Fluor 488. After 30 h. of incubation, EVs were collected and EU incorporation was analyzed by Flow Cytometry. (**b**) Distribution of the reads mapped to the *P. falciparum* genome according to the corresponding chromosomes. (**c**) Distribution of the reads mapped to the *P. falciparum* according to the size of the chromosome. (**d**) Types of *Plasmodium* genes in the EVs by RNA-Seq. (**e**) Extracellular vesicles contain a large number of Apicoplast tRNA and Mitochondrial rRNA. (**f**) RNA expression is different in EVs than mixed stage parasite expression pattern. (**g**) EV RNA composition is different that of iRBC. The expression levels shown by RNAseq of EV has no detectable correlation with that of steady state iRBC, showing that EV is not merely a smaller form of iRBC. Neither parametric (Pearson’s R) nor parametric correlations (Kendell’s tau) analysis detected correlation between EV and iRBC at any time points with correlation larger than 0.001. The X-axis shows the average steady state RNA expression levels.

Next, to investigate whether plasmodial RNA is released in the supernatant, we labelled newly transcribed RNAs in tightly synchronized iRBCs. Ring stage parasites were incubated with 5-ethynyl uridine (EU), which is incorporated into RNA during transcription. First, we monitored the cells for active transcription and, only iRBCs were positive for EU as measured by flow cytometry (Fig. 5a). Then, we collected the supernatants and freshly transcribed RNA was detected by flow cytometry in the supernatant with more than 80% of events being positive for freshly synthesized RNAs. Since RBCs are transcriptionally inactive, we assume that the labelled RNA is derived from the parasites (Fig. 5a). This experiment gave us strong confidence that EVs contain plasmodial RNA. In order to identify the plasmodial sequences, we aligned our RNA-Seq results generated previously to the *P. falciparum* genome (Plasmodium 6.1). We found that several plasmodial RNAs including tRNAs, rRNAs and mRNAs were present in EVs. In total, we identified 126 RNAs mapping to the *Plasmodium* genome of which 28 were identified in all the 3 samples (Supplementary Table 1). Most of the reads mapped to chromosome 1 (10.7%), chromosome 5 (33.1%), chromosome 7 (33.6%) and chromosome 13 (9.42%), only a minor amount mapped to mitochondrial and apicoplast DNAs (0.75 and 0.30%, respectively) (Fig. 5b). Then, we normalized by the length of the DNA, excluding mitochondrial and apicoplast DNAs since they are present in several copies per cell. Chromosome 1, 5 and 7 keep the highest number of mapped RNAs (Fig. 5c). Most of the RNA transcripts are derived from structural RNAs. There are a few putative novel ncRNAs, however their abundance is low. We have found mostly rRNA, tRNA and snoRNA in the three replicates from *Plasmodium* (Fig. 5d). In addition, a large amount of transcripts coding for proteins exported to the RBC cytosol such as RESA, ETRAMP and mRNAs from the PHIST family including Mal7P1.172 and PF0_0137 were present. Furthermore, EVs contain a large amount of apicoplast tRNAs^19^ and mitochondrial rRNAs (Fig. 5e), including tRNA-GLu-1, tRNA-Ser1, tRNA-Met1, tRNA-Pro1, tRNA-Arg1, tRNA-Trp1, tRNA-Arg3, tRNA-Cys1, pre-tRNA-Pr01, tRNA-SelCys1. Interestingly several RNAs were involved in drug resistance, such as PFE1150w (multidrug resistance protein 1), PFL1410c (ABC transporter, multidrug resistance-associated protein 2), PF13_0238 (Kelch-13).

We then compared the RNA EV expression profile with stage specific RNA profile of the blood stage and found that EV RNAs are mostly derived from genes with peak expression in merozoites and early rings. Very few RNAs are derived from other stages (Fig. 5f). Importantly, there was no direct correlation of the gene expression levels between iRBC and EV transcriptome (Fig. 5g).

### Plasmodial extracellular RNAs are associated with EVs

**Figure 6:**
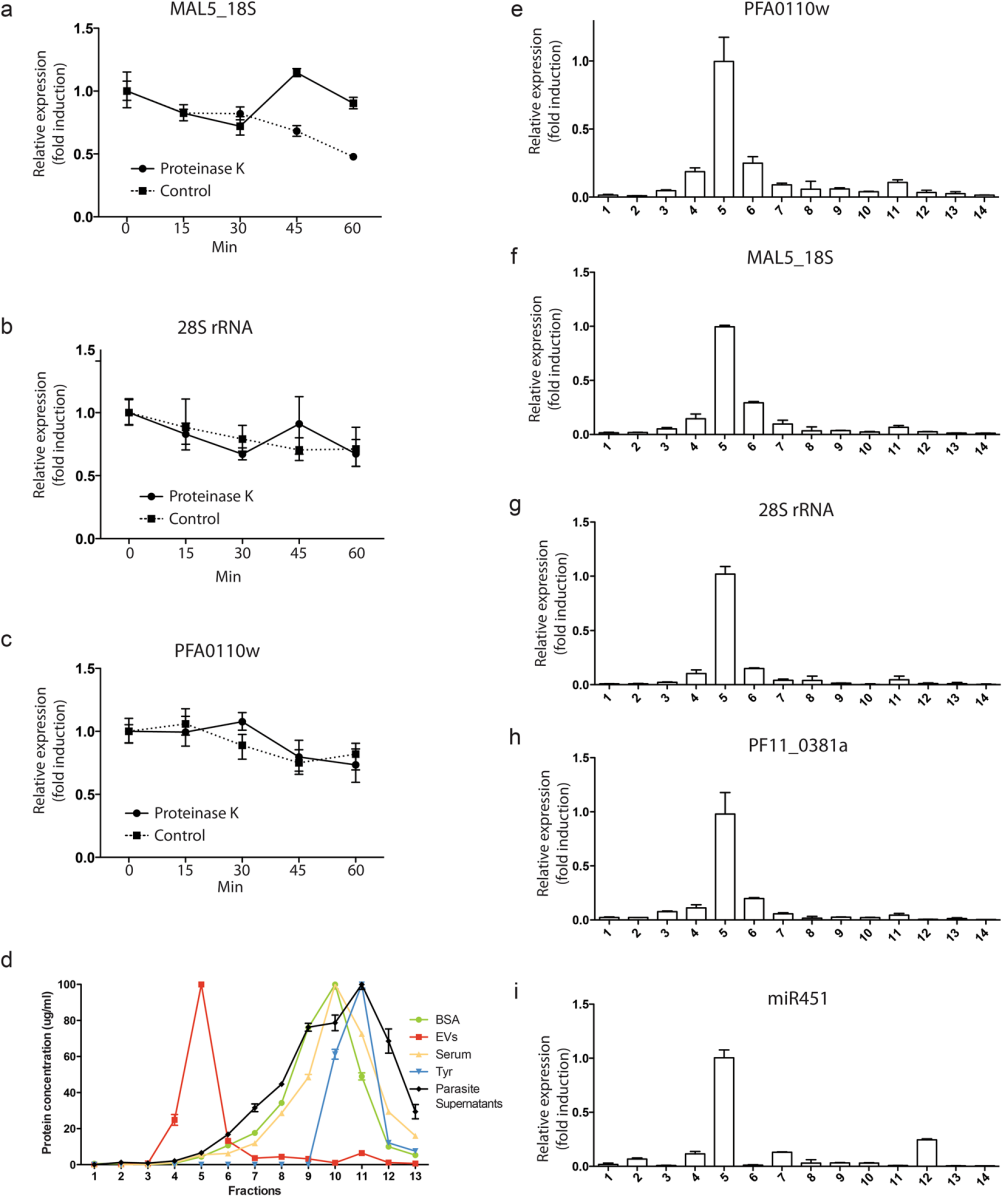
Plasmodial RNAs are contained inside the EVs. (**a**) The RNAs are protected from proteinase K digestion, EVs were treated with proteinase K (5 mg/ml, close symbols) or untreated (open symbol) at 55 °C. At times indicated aliquots were removed and the RNA was isolated and the expression of (**a**) MAL5_18S, (**b**) 28 S rRNAs, (**c**) PFA0110w, was analyzed by qPCR. The mean + /− s.d. of one representative experiments of three is shown. (**d**) Elution profile of conditioned media collected from iRBC culture as measured by absorbance at 280 nm. Points represent the mean +/− s.d. of three experiments performed in triplicate. (**e**) Fractions from conditioned media were assayed for (**e**) PFA0110w, (**f**) MAL5_18S, (**g**) 28 S rRNA, (**h**) PF11_0381a and (**i**) miR451 using absolute quantification by qPCR. The mean + /− s.d. of one representative experiment is shown (n = 3).

The extracellular RNAs found in the plasma can be associated with proteo-lipid complexes and the complexes containing RNAs might be co-purified with EVs during ultracentrifugation. To exclude the possibility that plasmodial RNA molecules originate from protein–lipid complexes present in our EV preparation or in the conditioned medium derived from *in vitro P. falciparum* iRBCs, we treated EVs with proteinase K^20^. In fact, proteolytic digestion of the protein complex stabilizing the plasmodial RNAs would release the RNAs and render them sensitive to degradation. However, RNAs protected inside a vesicular structure would not be expected to show sensitivity to proteolysis. We treated EVs with or without proteinase K at 55 °C. The samples were incubated for 0, 15, 30 and 45 min followed by RNA extraction and quantification by qPCR. In fact, MAL5_18S, 28 S rRNA and PFA0110w were protected from degradation inside EVs (Fig. 6a–c).

To further confirm that the plasmodial RNAs are associated with EVs, we performed size exclusion chromatography, which allows efficient separation of vesicles from protein lipid complexes that are smaller and elute later than vesicles. For this purpose, we cultured iRBCs and collected the conditioned supernatant after 48 hours. We isolated 14 fractions by chromatography and most of the proteins were eluted in the latest fractions (9–12), only a minor portion was eluted in the fraction 5 that corresponds to the EV fractions (Fig. 6d). We can assume based on our standards that most of the proteins in the supernatant are albumin, gamma-globuline. We then isolated total RNA including small RNAs from each isolated fraction and performed qPCR. As miR451a, all the tested plasmodial RNAs were detected in fraction 5, which corresponds to the EV fraction (Fig. 6e–i). Altogether these experiments demonstrated that *Plasmodium* RNAs are located in EVs.

### *Plasmodium* EVs deliver RNA cargo to human endothelial cells

**Figure 7:**
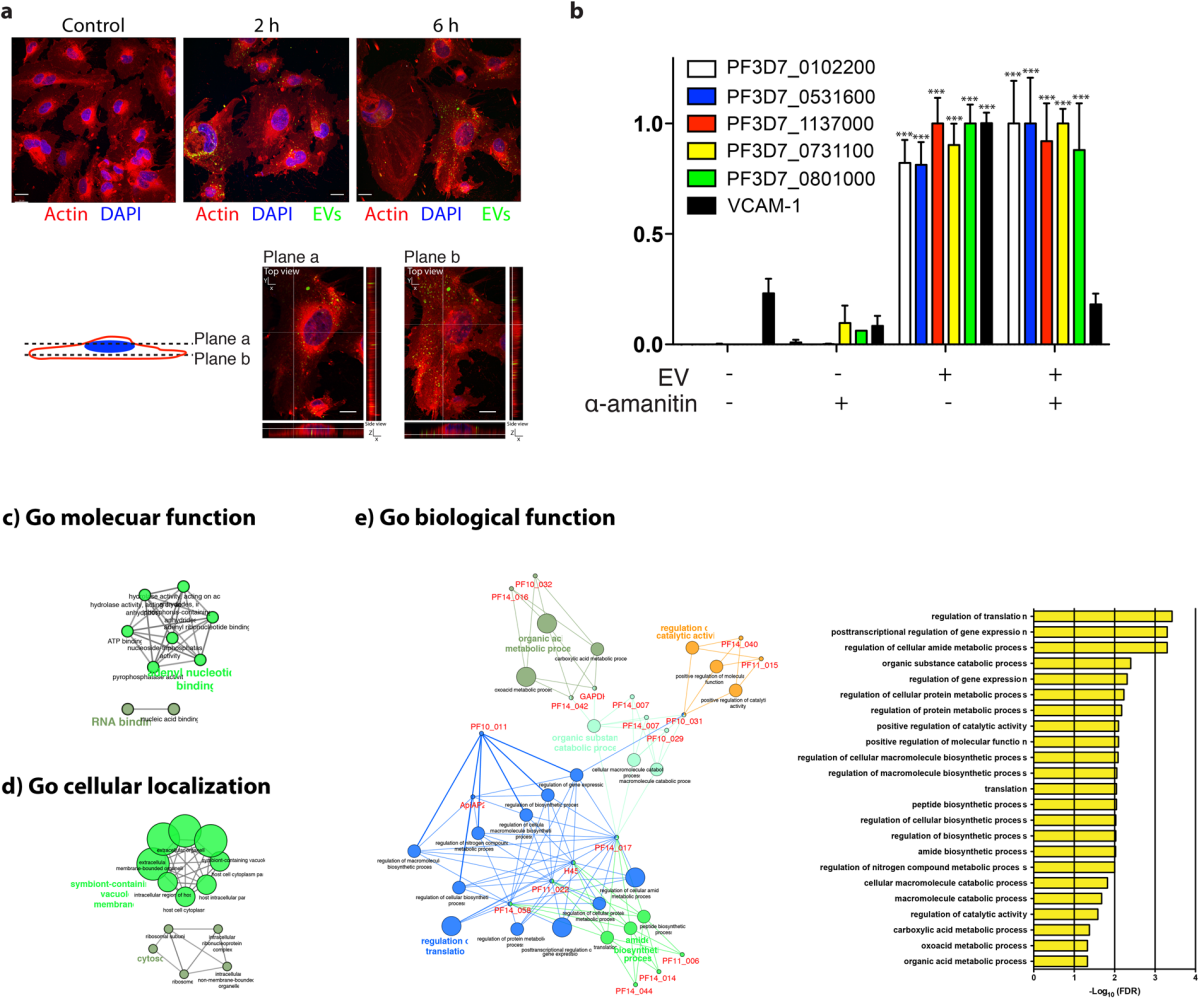
Transfer of plasmodial RNAs to endothelial cells via EVs. (**a**) Bone marrow endothelial cells (BMECs) were incubated with 100 μg of PKH67 fluorescently labelled EVs for 0 or 6 h. BMECs were stained for actin (phalloidin, red) and nuclei (DAPI, blue). Scale bar, 10 μm. (**b**) Endothelial cells were incubated with 100 μg of EVs for 12 hours. After isolation of their RNA, the transfer of plasmodial RNA was measured by qPCR. The average and S.D. of 3 experiments is shown. (mean ± s.e.m.; n = 3 experiments), ***P < 0.0001 versus control (Student’s t-test). (**c**) Functional classification of the identified *Plasmodium* RNAs in EVs by gene ontology (GO) analysis conducted using Cytoscape with the Cluego plugin. This highlights the regulatory function, which plasmodial RNAs may exert in the recipient cells. (**c**) GO molecular function (**d**) GO cellular localization (**e**) GO biological function.

To further understand the significance and potential function of the plasmodial RNAs in cellular communication, we tested their stability and potential transferability to endothelial cells. For this purpose, we incubated human bone marrow derived endothelial cells with (BMEC) EVs and noticed that the EVs were quickly taken up by the cells (Fig. 7a). We then monitored the transfer of RNAs by qPCR. No plasmodial RNA was detected in the absence of EVs. However, after a 12-hour EV-incubation the level of RNAs increased in endothelial cells. Therefore, the vesicles can act as a shuttle to transfer plasmodial RNAs to human endothelial cells. The inhibitor of the RNA Polymerase II and III (α-amanitin) was not able to inhibit the transfer, demonstrating that there was no active transcription of the *Plasmodium* genes in endothelial cells (Fig. 7b). To gain insight into the potential role of EV RNAs, we performed GO analysis. GO function analysis revealed that the RNA EVs were involved in RNA binding (Fig. 7c). Whereas GO cellular localization demonstrated that most of the RNAs are associated with the cytosol or vesicle membranes (Fig. 7d). Finally, the GO biological process showed that the RNAs are involved in translation regulation, regulation of gene expression and regulation of catalytic activity (Fig. 7e). Taken together these data further indicate a potential role of extracellular RNAs in regulation and cellular communication. Finally, we hypothesized that sequences that are identical between *P. falciparum* and human might have cross-regulatory function. From our RNA-Seq data, we identified several sequences that aligned to human and plasmodial genomes (Table 2).

**Table 2:** Reads mapping to *P. falciparum* and *Homo sapiens* genomes.

Location Human	Gene or comment
chr1:10,291,843-10,314,721	RNU6-1,2,7,8,9 intronic of KIF1B
chr1:91,344,238-91,430,371	HFM1 intronic
chr2:132,234,625-132,276,521	ANKRD30BL intronic (near MIR663B)
chr8:69,676,805-69,703,480	SLCO5A1 intronic
chr21:8,183,959-8,252,641	NR_038958 and 28 S RNA intronic (near MIR3648 and MIR663A)
chr21:8,393, 229-8,410,398	NR_038958 intronic and 28 S RNA exonic
chr21:8,429,258-8,463,598	28 S RNA 45 S RNA exonic
**Location Plasmodium**	**Gene or comment**
many	nearly all rRNA genes
Supercontig_1.3:841,093–845,256	T complexe protein PFC0900w
Supercontig_1.4:520,965-530,102	tRNA-Glu1
Supercontig_1.7:716,587–734,017	tRNA-Asp1
Supercontig_1.11:126,943-135,040	near PF11_0040-1 (3′utr?)
Supercontig_1.11:1,462,718-1,466,266	near PF11_0383-1 (promoter?)
Supercontig_1.12:582,683-593,330	near PFL0665c (promoter?)
Supercontig_1.13:1,568,006-1,594,376	tRNA-Pro1
Supercontig_1.13:1,632,229-1,633,112	snRNA-1 U6
Supercontig_1.13:2,793,179-2,807,332	several unknown function proteins (MAL13P1.430, 435, 440, 455, 460)
Supercontig_1.14:1,670,919-1,676,282	PF14_0390 unknown function protein

## Discussion

To date, a detailed analysis of the nucleic acid composition of malarial EVs has been lacking and only the host miRNA composition has been determined^7^. This study is the first systematic description of EV small-RNAs in *P. falciparum* iRBCs. With our approach, we found that EVs contain human, as well as plasmodial RNAs with potential regulatory functions. Interestingly the EVs were able to deliver plasmodial RNAs to endothelial cells suggesting a potential role in pathogen-host cellular communication. Furthermore, the full characterization of the EV RNA content offers a large number of promising new biomarkers for the diagnosis and the prediction of the progression of the disease.

Transfer of functional RNAs between cells via EVs is a universal mechanism of cellular communication^21^. Here, we identified several small RNAs with regulatory function. The host miRNAs were the most abundant RNA species detected in the EV samples. miRNAs are crucial in regulating posttranscriptional gene expression^22^. Recently miRNAs were shown to shuttle from cell to cell via EVs to mediate cellular communication^23^. In the context of malaria, we have previously shown that miR451a regulates cellular functions after transfer to endothelial cells^7^. Here, in addition to miR451a, we identified miR486-5p and miR92a-3p as the highest expressed human miRNAs. All the three miRNAs are expressed in RBCs and play an essential role in their maturation^24,25^. They regulate erythropoiesis by inhibiting proliferation or differentiation, protecting erythroid cells from oxidative stress or controlling iron transport and metabolism. However, these miRNAs have a function beside their role in RBC maturation^26,27^. There is only little overlap between the genes targeted by the EV miRNAs. However, the miRNAs target the same functional pathways. This raises the possibility that the transferred EV miRNAs (including mir451a, mir486-5p and mir92a-3p) might act synergistically to target multiple genes part of the same pathways involved in malaria infections and pathogenesis. In fact, our analysis of the KEGG pathways showed that iRBC-EV miRNAs targeted pathways involved in cell proliferation and adherence junction. Although little is known about the specific role of iRBC EVs in Cerebral Malaria pathogenesis, the concentration of EVs derived from RBCs, platelets, endothelial cells and leucocytes are markedly elevated in patients and there is a clear association between EV concentration and pathogenesis. More direct evidences for a role of EVs are coming from the rodent model of malaria. The reduction of EVs in transgenic mice or with drugs confers protection against severe disease and cerebral malaria. Furthermore, EVs isolated from mice with malaria are potently activating macrophages^4,5,28–33^. It remains to be demonstrated whether EV RNAs derived from different cellular sources can act synergistically to modulate the pathogenesis. In addition to miRNAs, we identified numerous host tRNA fragments. This is in agreement with other studies that found tRNAs in immune cells derived EVs^8^, in semen EVs^9^ and in EVs secreted by pathogens such as *Trypanosoma cruzi*^34^ and leishmania^35^. While tRNAs, which are 73–90 nt in length are components of the translational machinery, a functional role in gene regulation has been proposed for tRNA fragments. In our samples the most abundant tRNA (tRNA-Glyc) was derived from a 5′ and 3′ halves. In addition, we found 5′ end tRNA fragments, predominantly 30–34 nt tRNA halves derived from tRNA-Lys and tRNA-Phe. These derivatives of tRNAs are not degradation products but are specific cleavage products that function in pathophysiological conditions^36^. During oxidative stress, angiogenin cleaves the full-length mature tRNAs and produces 5′ and 3′ halves that are 30–40 nt in length^37^. The 5′ ends of these tRNA halves inhibit translation by displacing components of the translation initiation complex^38^. tRNA fragments are heterogeneous in size, nucleotide composition, biogenesis and function. Besides tRNA halves, shorter tRNA-derived fragments (tRFs) in the size range of 13–30 nt have been described in organisms spanning all domains of life. These RNAs can be both constitutively generated and produced in the context of stress. Although angiogenin is not detected in EVs^3^, it is expressed in RBCs^39^. The oxidative environment that triggers the parasite in the RBCs might favor the generation of tRFs that are then secreted via EVs. While, the role of tRNA in cellular communication is unknown, their biological function range from translation control, over RNA silencing, to regulating apoptosis^40,41^. Similar to earlier descriptions of exosomal and circulating tRNAs, we identified primarily 18–35 nt fragments in our analysis, supporting the potential processing of tRNA to miRNA-like molecules^8,9^.

While numerous piRNAs and snoRNAs are found in the circulation, their significance in biological processes or diseases is unknown^42,43^. In our samples, the piRNAs were almost as abundant as the miRNAs. Canonical piRNAs are defined as small RNAs bound to PIWI proteins^44^. Recently, functional roles for piRNA beyond transposon silencing have emerged including genome rearrangement^45^, epigenetic regulation^46^ regulation of mRNA^47^ and lncRNAs^48^. piRNAs target the 3′UTR of several gene products that have to be degraded by acting similarly to miRNAs. Therefore, piRNAs may have potentially an important role in gene regulation and cellular communication.

Moreover, snoRNAs can give origin to shorter miRNA-like RNAs capable of regulating translation due to complementary interactions with mRNA^49^. The importance of snoRNAs in biological processes is illustrated by the absence of SNORD116 (SNO1354) in the Prader-Willi syndrome (PWS), a severe hereditary disease associated with obesity, mental retardation, and some other symptoms^19,50^.

PIR13807, which was the most abundant piRNA in our study, as well as PIR58593 were identified in the plasma of a large cohort of individuals^42^. SNO1408 (SNORD10) and SNO1257 (SNORD114) were also identified in plasma of healthy individuals^42^.

In addition, in our samples we found Y RNAs. Y RNAs are components of ribonucleoproteins (RNPs) complexed with Ro60 and La proteins. Although full length Y RNAs have a size of approximately 100 nt, high-throughput RNA sequencing has revealed that small Y RNA fragments, 22–36 nt, are also highly abundant in cells, tissues and body fluids including in EVs of humans, as well as in a range of tumors^9,51,52^.

We found that RNY4 is the most abundant in EVs. Interestingly Y RNA-derived fragment is the most abundant fragment in plasma and serum in healthy patients^52^, as well as in EVs found in semen^9^.

Altogether, the function of the extracellular tRNAs, piRNAs, snoRNAs and Y-RNAs is currently unknown. Interestingly, only the processed forms of the RNAs were found in the EVs. Therefore, it can be speculated that the small RNAs are specifically targeted to the EVs. It is an intriguing possibility that small RNAs could mediate intercellular physiological signals to the hosts or between parasites.

While host small RNAs were the most abundant, we also found *Plasmodium* small RNAs such as tRNA, snoRNAs. In addition, we found *Plasmodium* mRNAs coding for proteins with gene regulatory properties such as the Alba proteins. The Alba proteins bind to RNA and fine tune the translation of RNAs linked to invasion^53^. We also identified *Plasmodium* RNAs with regulatory properties such as PFC0425w (Plant Homeodomain (PHD) finger protein) and PFF1185w (Smarca-related protein) which have chromatin-mediated gene regulation, as well as transcription factor with an AP2 domain (PF08_0074). It remains to be investigated whether the parasite mRNAs are translated in the recipient cells.

Parasite resistance to antimalarial drugs is an increasing problem in the fight against malaria^54^. Surprisingly, EVs carry host and *Plasmodium* RNAs involved in drug resistance. The vtRNAs are part of a large cellular ribonucleoparticles called vaults, which are implicated in chemotherapy resistance and have been implicated in the regulation of several cellular processes including transport mechanisms^55^, signal transmissions and immune responses^56^. vtRNAs are upregulated in several tumors where they mediate drug resistance, which can be conferred either by direct binding to chemotherapeutic compounds^57,58^ or by the regulation of expression of multidrug-associated proteins^59^. Whether vtRNA1-1 plays a role in acquiring drug resistance to malaria therapies is an intriguing possibility. It might act by binding to a drug to participate in the export of malaria therapeutics^58^.

Furthermore, we identified several parasite-derived mRNAs coding for proteins involved in drug resistance such as PFE1150w (multidrug resistance), PFL1410c (ABC transporter, multidrug resistance-associated protein 2). As well as PFKELCH13, which is involved in artemisinin resistance^60^.

After alignment of the RNAseq reads against the plasmodial genome, we found that EVs contain plasmodial RNAs. Most of the transcripts are derived from structural RNAs, including a few putative novel ncRNAs. We demonstrated the presence of PF11_0381a snoRNA U2 spliceosome in EVs and its EV-mediated transfer to endothelial cells. In addition, some plasmodial mRNAs were identified. Interestingly a large number of the plasmodial mRNAs are coding for exported proteins^61^, such as PHIST B and PHIST C, PFA0110w, PFD1170c, MAL7P1.171, MAL7P1.172, PF08_0137. Why and how plasmodial RNAs are exported to EVs is not clear. However, the large percentage of small RNAs with potential gene regulatory function suggests they might be involved in cellular communication. The recent discovery of pathogen derived regulatory RNAs in several EVs suggests that regulatory RNAs may function in interspecies regulation involving microbial RNAs and host genes. It is possible that *P. falciparum* transfer its own RNAs via EVs to human cells to regulate gene expression to its own advantage. Some outstanding questions remain such as how *P. falciparum* targets specifically these RNAs to EVs or are the transferred RNAs transcribed in the target cells, either host cells or parasites.

While our and other results suggested that iRBCs secrete about 15 more EVs than uninfected RBCs, our current results with the transfer of plasmodial RNA showed that our preparations may contain more than 80% EVs derived from iRBCs^3,4^.

Taken together our data identified for the first time the RNAs species contained in EVs. We found host as well as parasite derived RNAs. Most of the RNAs have gene regulatory functions and might therefore be involved in cellular communication. In addition, we found several RNAs involved in drug resistance and aspect that will require more investigations. Finally, we also found *Plasmodium* derived RNAs. Therefore, our study offers a wide range of new biomarkers as well as new potential pathways that can be targeted by drugs.

## Materials and Methods

### Cell culture of parasites

The *P. falciparum* strain 3D7 was used for this study. Parasites were kept in fresh type 0 + human red blood cells, suspended at 4% hematocrit in HEPES-buffered RPMI 1640 containing 10% (w/v) heat inactivated human serum, 0.5 ml Gentamycin, 2.01 g sodium bicarbonate and 0.05 g Hypoxanthine at pH 6.74. Prior to culture, the complete medium was depleted from extracellular vesicles and debris by ultracentrifugation at 100000 g for 1 hour. The parasite cultures were maintained in a controlled environment at 37 °C in a gassed chamber at 5% CO_2_ and 1% O_2_.

### Synchronization of parasites

In order to obtain highly synchronized parasite cultures we performed a combination of Percoll and sorbitol. Parasites at 42–45 hours post-invasion were purified using a 70% Percoll. Fresh blood was added to the isolated schizonts. After 8 hours, 5% sorbitol was used to eliminate the remaining schizonts to yield highly synchronized rings.

### Purification of EVs

EVs from iRBCs were isolated from cell culture supernatants as described^3^. In brief, cell culture supernatants of *Plasmodium falciparum*-infected RBCs were collected. Cells and cellular debris were removed from the supernatant by centrifugation at 600 g, 1600 g, 3600 g and finally 10000 g for 15 min. To further concentrate the EVs, the supernatant was filtered through a Vivacell 100 filter (100 kDa molecular weight cut off; Sartorius). Then, the concentrated supernatant was pelleted at 100000 g, the pellet resuspended in PBS and layered on top of a 60% sucrose cushion and spun at 100000 g for 16 hours. The interphase was collected and washed with PBS twice at 100000 g for 1 hour to yield EVs.

### Isolation of RNA and gene expression studies in BMEC-1

Total RNA was isolated from EVs and BMEC-1 using miRNeasy kit (Qiagen, Hilden, Germany). The concentration and integrity of total RNA was measured using a NanoDrop-1000 spectrophotometer (Thermo Scientific, Wilmington, DE). Size distribution was determined by Agilent 2100 Bioanalyzer with the Agilent Small RNA Chip (Agilent Technologies, Santa Clara, CA).

For Plasmodial RNAs, reverse transcription reactions were performed with 1 μg total RNA using M-MLV Reverse Transcriptase kit (Promega) after DNase I treatment (Invitrogen).

Mature miR-451a and selected miRNAs were detected by quantitative RT-PCR (qRT-PCR) using TaqMan MicroRNA Reverse Transcription Kit (Applied Biosystems). Small nuclear RNA U6 (RNU6) (for miR-451a) and 18 S rRNA and EF1 (for mRNAs) were used as reference genes for relative quantitation using the 2^^− Ct^ method.

### Metabolic labeling of RNA and imaging

Ring stage parasites at a parasitemia of about 5% (3D7), (15 hours post-invasion) were incubated at a final concentration of 0.5 mM 5-ethynyl uridine (EU) for 30 h. EVs were isolated from the conditioned medium as described above with exception of the sucrose gradient. The cells and EVs were subsequently fixed with 4% para-formaldehyde and permeabilized with 0.1% Triton-X-100. EU incorporated EV RNA was detected using Click chemistry according to the manufacturer’s protocol (Invitrogen, C10329) and nuclei were counterstained using Hoechst 33342 and analyzed with a MACSQuant VYB flow cytometer. Flow cytometry data acquisition was performed using FlowJo X (Tree Star, Ashland, OR).

### Endothelial cell culture

A semi-immortalized human bone marrow derived endothelial cell line (BMEC-1)^62^ was cultured in Endothelial Cell Growth Medium MV (Promocell) supplemented with 10% fetal bovine serum (Cedarlane, Burlington, Canada) and maintained at 37 °C under 5% CO_2_.

EV internalization assays with BMEC-1. Purified EVs were labelled with PKH67 red fluorescent labelling kit (Sigma-Aldrich) and incubated with BMECs for 12 h before washing cells three times to remove unbound EVs. For uptake inhibition experiments endothelial cells were co-incubated with different compounds, as described in Fig. 5. Cells were fixed, permeabilized and stained for F-actin with CF594 (Biotium) and with the nuclear dye DAPI (Sigma-Aldrich). Leica TCS SP5. Epifluorescence was performed on a Axiovert 200 M microscope (Carl Zeiss. Germany) equipped with a camera (Orca charge-coupled device; Hamamatsu Photonics, Japan) using a 40 Plan-Neofluar (NA 1.3) oil immersion objective. Intensity analysis was performed with the Axiovision 4.6.3 software. Background subtraction was performed for each field. Confocal microscopy was performed on a Zeiss LSM 510 Laser Scanning Microscope using a 63 Plan-Neofluar water-immersion objective.

### Size exclusion chromatography

Experiments to determine possible presence of serum-derived Ago2-miRNA complexes were performed essentially as described^7^.

Briefly, sephacryl S-500 resin (GE Healthcare) was packed in a chromatography column (0.9 Å~ 30 cm, 19.1 mL bed volume). Before injection, the column was equilibrated with 25 mL of PBS solution at 0.5 mL/min at room temperature. The column was injected with 0.5 mL of undiluted fresh serum or purified EVs and eluted at 4 °C for approximately 1 h with PBS solution (pH 7.4) at a flow rate of 0.5 mL/min. A total of 25 fractions of 1 mL each were collected. Fractions were stored at 4 °C before use. Protein molecular weight standards included BSA (67 kDa; GE Healthcare) and tyrosine (0.181 kDa; Sigma-Aldrich).

### Gene ontology analysis

ClueGO was used to find over-represented GO terms in the categories “biological process”, “cellular component” and molecular function”. Benjamini-Hochberg correction was performed for multiple testing-controlled P values. The GO analysis was conducted using Cytoscape 3.2.1 software^63^ with the ClueGO^64^ plugin. The GO analysis was conducted using a two-sided hypergeometric test with Bonferroni correction. The GO term levels were from five to ten. The minimum number of genes to form a cluster was set at three, while the minimum percentage of genes covered by our data set against the database was set at 7%. The rest of the settings were left as defaults.

### RNA-Seq

Each sequencing library was constructed from 2 ng of isolated and treated plasma RNA. All libraries were uniquely bar-coded with index primer for multiplexing into sequencing lanes. The small RNA libraries were prepared and amplified using the NEBNext small RNA Library Prep Set (New England BioLabs, Ipswitch, MA, USA) following manufacturer instruction. The amplified libraries were resolved on a 10% Novex TBE gel (Life technologies) for size selection and the 140 to 160 nucleotide bands that correspond to adapter-ligated constructs derived from the 21 to 40 nucleotide RNA fragments were excise and recovered in DNA elution buffer. The average size distribution of each library was determined using Agilent Bioanalyzer with High Sensitivity Chip Kit (Agilent, Santa Clara, CA, USA) and quantified on ABI 7900HT Fast RT-PCR instrument using the KAPA Library Quantification kit according to the manufacture’s protocol (Kapa Biosystems, Woburn, MA, USA). Each library was adjusted to final concentration of 2 nM, pooled, and sequenced on an Illumina HiSeq. 2000 or MiSeq sequencer for single read 50 cycles at the Center for Cancer Computational Biology at Dana-Farber Cancer Institute.

### Sequence Analysis

The BCL files were de-multiplexed using CASAVA v1.82, and the adaptor sequences within the read sequences were trimmed by FastX-Toolkit (http://hannonlab.cshl.edu/fastx_toolkit). The processed sequences were filtered for small RNAs greater than 16 nucleotides in length. The sequences were then aligned, quantified and annotated using sRNABench 1.0 pipeline^65^. Briefly, the pipeline implemented hierarchical sequence mapping strategy that first mapped and remove spike-in library, contaminants, and rRNA before sequentially mapped to known mature miRNA, tRNA, snoRNA and piRNA onto the human genome sequence (hg19) using Bowtie2^66^ with parameters that allow for 1 mismatch in seed alignment (-N 1), try two set of seeds (-R 2), and set the length of seed substrings to be 16 (-L 16). Mapped small RNA species was quantified to read counts and normalized to RPM as described in sRNABench. Detected species were mapped to mature miRNA only and not precursor miRNA. Reads derived from microRNA with multiple copies in the genome were summed together, and read counts from sample duplicates were aggregated by mean for where it is applicable. All statistical analysis was performed using R version 3.2.

## Electronic supplementary material

Supplementary figure 1
