# Supplementary material for: Malaria infected red blood cells release small regulatory RNAs through extracellular vesicles
Source: Sci Rep. 2018 Jan 17;8:884. doi: 10.1038/s41598-018-19149-9 (PMC5772623; doi:10.1038/s41598-018-19149-9)
Supplement: Supplementary file 1 — Supplementary figure 1 [file 41598_2018_19149_MOESM1_ESM.pdf]

Supplementary Figures 1-3

Malaria infected red blood cells release small regulatory RNAs through extracellular vesicles

Kehinde Adebayo Babatunde[1#], Smart Mbagwu[1#], María Andrea Hernández-Castañeda[1#], Swamy R. Adapa[2], Michael Walch[1], Luis Filgueira[1], Laurent Falquet[1], Rays H. Y. Jiang[2], Ionita Ghiran[3], Pierre-Yves Mantel[1]*

[#]Contributed equally

[1]Department of Medicine, University of Fribourg, 1700 Fribourg, Switzerland
[2]Department of Global Health (GH) & Center for Drug Discovery and Innovation (CDDI), College of Public Health, University of South Florida, Tampa, FL 33612 USA
[3]Division of Allergy and Infection, Beth Israel Deaconess Medical Center, Boston, MA 02115 USA
*Corresponding author: Pierre-Yves Mantel (Pierre-Yves.mantel@unifr.ch)

Supplementary Figure 1

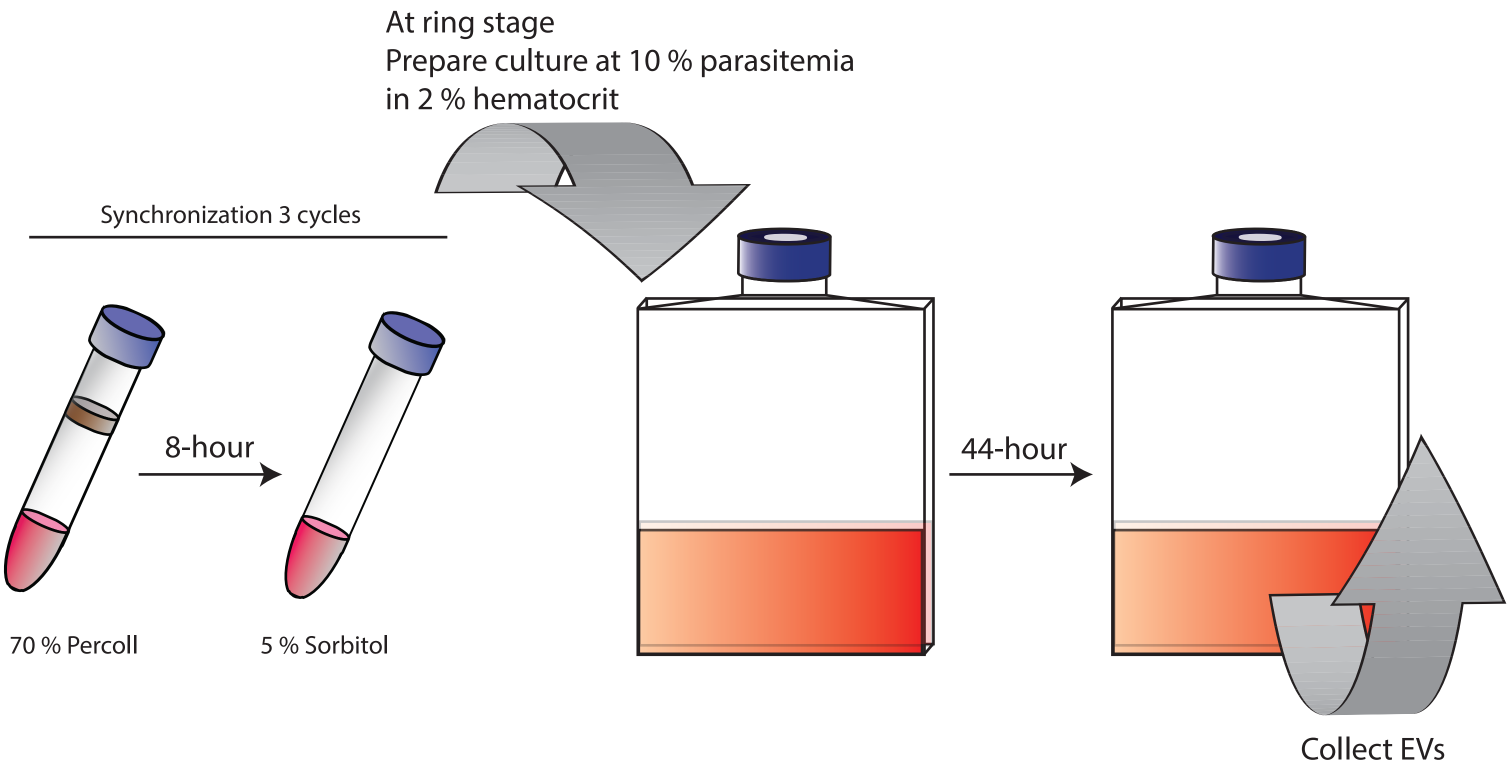

Supplementary Figure 2

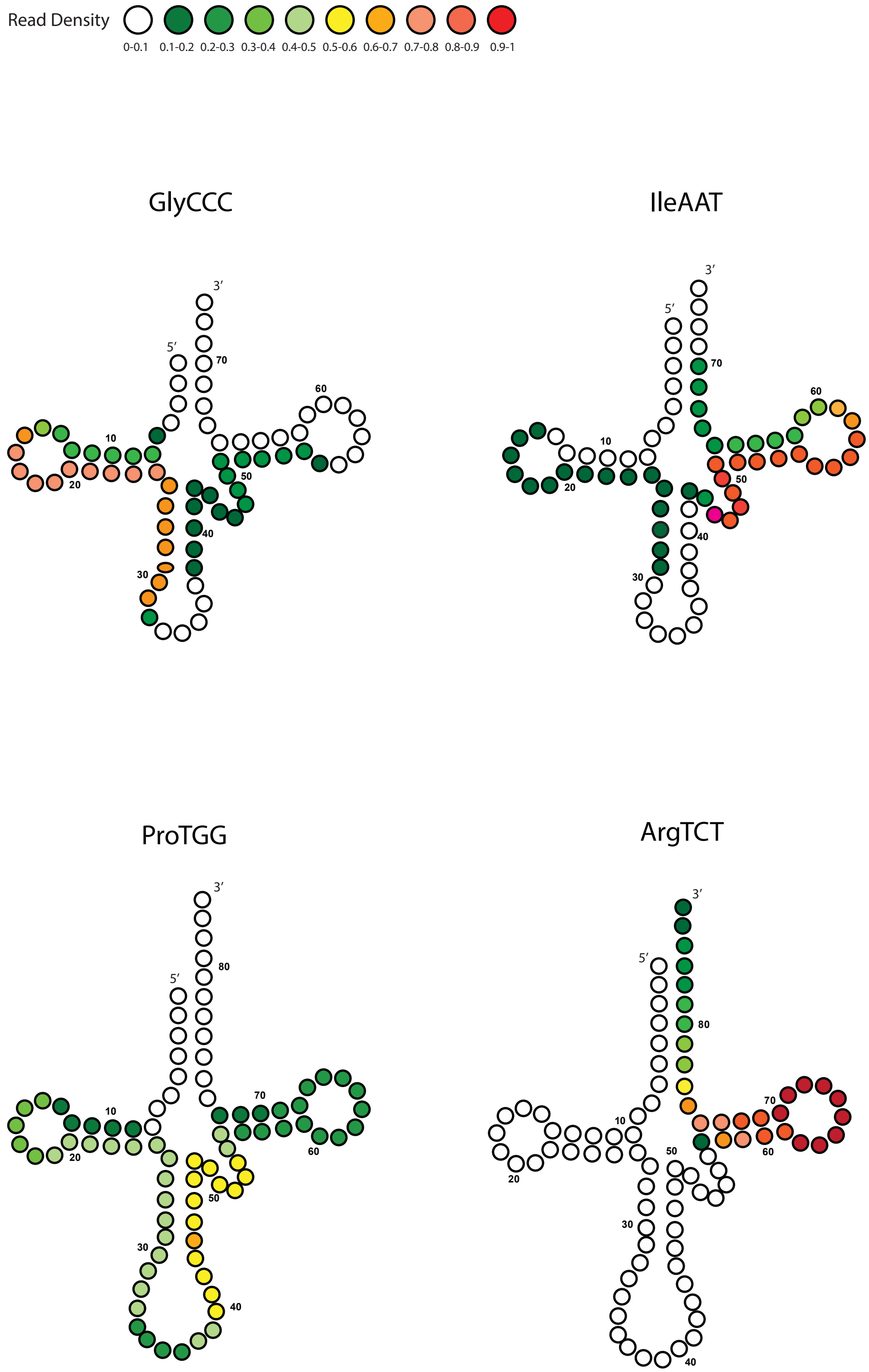

Supplementary Figure 3 Full scans of uncropped blots presented in the main figures

Fig. 1c

Stomatin

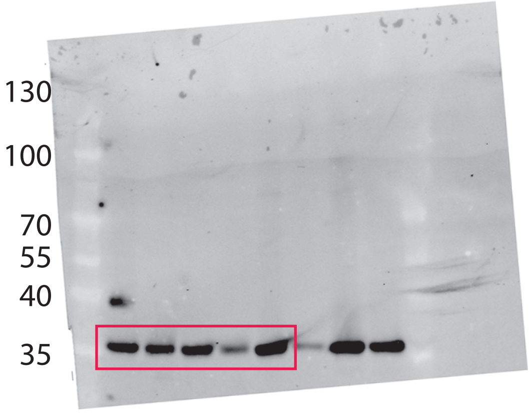

Bip PF3D7_0917900

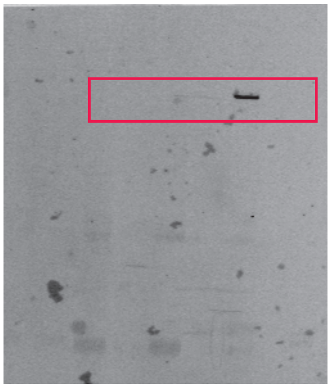

RESA PF3D7_0102200

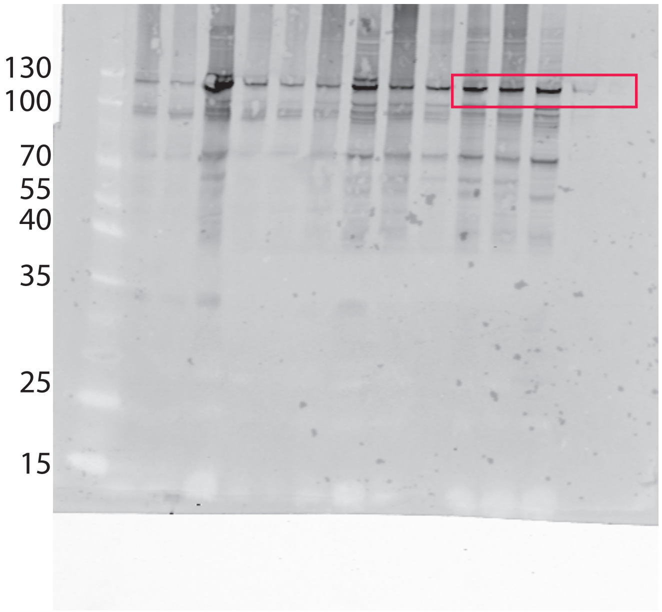

Supplementary Figure S1:
The parasites were synchronized by a combination of Percoll and sorbitol treatments. Late stage parasites were isolated by a 70% percoll gradient. After 8-hour incubation the cultures were subjected to 5% sorbitol treatment to kill the remaining late stage parasites. This synchronization protocol was repeated over 3 complete cycles to yield highly synchronized cultures. For each replicate a total of 1-liter culture at a parasitemia of 10 % in 2 % hematocrit were started. The cells were washed shortly after egress and diluted in fresh blood to give a parasitemia of 10%. Fresh medium depleted from EVs was added and forty-four hours after the medium was collected for EV purification.”

Supplementary Figure S2:
Based on the small RNA-seq results, only tRNA fragments were identified in EVs. Shown are examples of tRNA structures for each species depicting the boundaries of the identified tRNA fragment. The read density at each position is shown as a heatmap (red: high coverage; transparent: no coverage). The structure for GlyCCC, IleAAT, SerACT, ProTGG and ArgTCT are depicted.

Supplementary Table 1:
RNA-Seq expression analysis. Shown are normalized RNA-Seq expression values for *P. falciparum* RNAs in the 3 samples. The Fragments Per Kilobase of transcript per Million mapped reads (FPKM) was used to measure the expression level of the RNAs.
